# Tumour associated lymphocytes in the pleural effusions of patients with mesothelioma express high levels of inhibitory receptors

**DOI:** 10.1186/s13104-018-3953-x

**Published:** 2018-12-05

**Authors:** Jonathan Chee, Mark W. Watson, Abha Chopra, Bella Nguyen, Alistair M. Cook, Jenette Creaney, Willem J. Lesterhuis, Bruce W. Robinson, Y. C. Gary Lee, Anna K. Nowak, Richard A. Lake, Alison M. McDonnell

**Affiliations:** 1 National Centre for Asbestos Related Diseases, School of Medicine and Pharmacology, University of Western Australia, Nedlands, Western Australia Australia; 2 Institute for Immunology and Infectious Diseases, Murdoch University, Murdoch, Australia; 3 Department of Medical Oncology, Sir Charles Gairdner Hospital, Nedlands, Western Australia Australia; 4 Department of Respiratory Medicine, Sir Charles Gairdner Hospital, Nedlands, Western Australia Australia; 5 Institute of Respiratory Health, School of Medicine, University of Western Australia, Sir Charles Gairdner Hospital, Nedlands, Western Australia Australia

**Keywords:** Mesothelioma, Pleural effusion, T cell receptor

## Abstract

**Objective:**

Pleural effusion (PE) is a common feature of malignant pleural mesothelioma. These effusions typically contain lymphocytes and malignant cells. We postulated that the PE would be a source of lymphocytes for analysis of tumor immune milieu. The aim of this study was to compare the phenotype and T cell receptor usage of pleural effusion T cells with paired concurrently drawn peripheral blood lymphocytes. We used multi-parameter flow cytometry and high-throughput T cell receptor sequencing to analyse peripheral blood and pleural effusion mononuclear cells.

**Results:**

Both CD8^+^ and CD4^+^ T cells from effusion showed increased expression of T cell inhibitory receptors PD-1, LAG-3 and Tim-3 compared to blood. Comprehensive T cell receptor sequencing on one of the patients showed a discordant distribution of clonotypes in the antigen-experienced (PD-1^+^) compartment between effusion and blood, suggesting an enrichment of antigen specific clonotypes in the effusion, with potential as an immunological response biomarker.

## Introduction

Malignant mesothelioma originates from mesothelial cells lining the pleura. Most patients have a history of asbestos exposure, present with advanced disease and receive palliative chemotherapy only. The median survival for patients treated with cisplatin and pemetrexed is around 12 months [1] and most patients die from their disease.

The success of immunotherapy targeting checkpoint inhibitory T cell receptors in a variety of cancer types, has seen the anti-tumour immune response re-emerge as a promising prognostic marker, therapeutic target and a potential biomarker of response for cancer immunotherapy [2]. Such therapies are under clinical investigation for the treatment of patients with mesothelioma [3], and understanding T cell responses in these patients is crucial for improving the efficacy of immunotherapy.

The prognostic significance of tumour infiltrating lymphocytes (TILs) is established for many cancer types including mesothelioma [4]. Longitudinal characterisation of T cell phenotype and T cell receptor (TCR) usage at the tumour site could help us to understand how to promote effective anti-tumour immunity. However, serial analysis of immune changes at the tumour site is rarely feasible in clinical practice as tumour biopsies are invasive. Malignant pleural effusions (PE), a recurrent and common complication of mesothelioma, are regularly drained as a palliative measure and can be sampled serially [5]. PE is an abnormal accumulation of fluid in the pleural space with T lymphocytes the predominant immune cells [6]. Given that malignant PE are intimately associated with mesothelioma, they form an important part of the local tumour environment and as such, immune events in the PE may serve as surrogate markers of the intratumoural milieu. We characterized T cell phenotype in PE and peripheral blood in 9 patients with mesothelioma, and compared the T cell repertoire between PE and peripheral blood of a selected patient.

## Main text

### Materials and methods

#### Sample collection and processing

This study was approved by the site Human Research Ethics Committee and the patient provided written informed consent for collection and analysis of biological samples. Blood was collected into a heparinized Vacutainer^®^ (BD Diagnostics) and peripheral blood mononuclear cells (PBMC) isolated according to manufacturer’s instructions. A 50 ml PE sample was collected. Ficoll density centrifugation was used to isolate PE mononuclear cells (PEMC). Cells were stored in liquid nitrogen until analysis.

The nine participants in the current study had a histologically or cytologically confirmed diagnosis of malignant pleural mesothelioma. Participants could have any stage or duration of disease; prior treatment with chemotherapy or radiotherapy was allowed. Effusions from patients with prior talc pleurodesis or pleural infection were excluded from this study.

#### Flow cytometry

Thawed cells were labelled with antibodies: CD14-APC-eFluor^®^780 (61D3), CD19-APC-eFluor^®^780 (HIB19), LAG-3-PE (3DS223H) (eBioscience); CD45-BUV395 (HI30), CD3-BV510 (UCHT1), CD4-BV711 (OKT4), CD8a-FITC (RPA-T8), PD-1-PerCP-Cy5.5 (EH12.2H7), PD-1-PECy7 (EH12.2H7), Tim-3-APC (F38-232), CD45RA-BV785 (HI100) (BioLegend). Dead cells were excluded using Viability Dye eFluor™780 (ThermoFisher). Samples were acquired on a LSR Fortessa (BD Biosciences) and analyzed using FlowJov10 (Treestar).

#### TCR sequencing

Cells were sorted on CD3, CD4, CD8 and PD-1 expression with a BD Influx™ (BD Biosciences). DNA was extracted from sorted cells using QIAmp DNA Kit (Qiagen). DNA sequences were amplified with a Human TCRβ ImmunoSeq Kit (Adaptive Biotechnologies). Sequencing was performed on a MiSeq (MS-102-3001, Illumina) and data was analyzed using the Adaptive ImmunoSeq suite. Each TCR clonotype is defined as a unique TCRβ nucleotide sequence. Differential abundance of T cell clones were compared as previously described [7]. The diversity of each sample was assigned a clonality score [8], whereby each score varied from 0 (each clone appearing once) to 1 (monoclonal sample).

### Results

#### Malignant effusions have proportionately more T cells that express inhibitory receptors, compared to blood

Pleural effusion mononuclear cells (PEMC) were contemporaneously collected with peripheral blood mononuclear cells (PBMC) from 9 male patients with pleural mesothelioma. Patient characteristics are shown in Table 1. Biological samples were analyzed by multi-parameter flow cytometry. The CD3^+^ T cell component of both PE and peripheral blood demonstrated similar proportions of the CD8^+^ and CD4^+^ subsets (blood vs. effusion, CD4^+^ T cells: 71.9% ± 10.1% vs. 70.3% ± 12.5%; CD8^+^ T cells: 22.2% ± 9.7% vs. 23.3% ± 9.6%).

**Table 1:** Patient characteristics at the point when effusion and blood samples were taken

Patient no.	Age (at sample date)	Sample time from diagnosis (days)	Histology	Prior treatment	Indwelling pleural catheter (IPC)/thoracentesis	Effusion vol. (ml)	Cell concentration in effusion (× 10^7^/100 ml)
1	76	335	Epithelioid	No	IPC	1050	3.60
2	66	145	Epithelioid	No	Thoracentesis	535	8.47
3	80	403	Epithelioid	Carboplatin/pemetrexed	Thoracentesis	200	3.02
4	61	705	Epithelioid	No	IPC	700	9.00
5	58	250	Epithelioid	Cisplatin/pemetrexed	IPC	300	2.16
6	59	50	Epithelioid	No	Thoracentesis	950	3.90
7	76	14	Not specified	No	IPC	1450	2.21
8	68	68	Epithelioid	No	Thoracentesis	1080	2.22
*9	72	389	Not specified	No	IPC	120	2.99

*Patient samples underwent T cell receptor sequencing

**Fig. 1:**
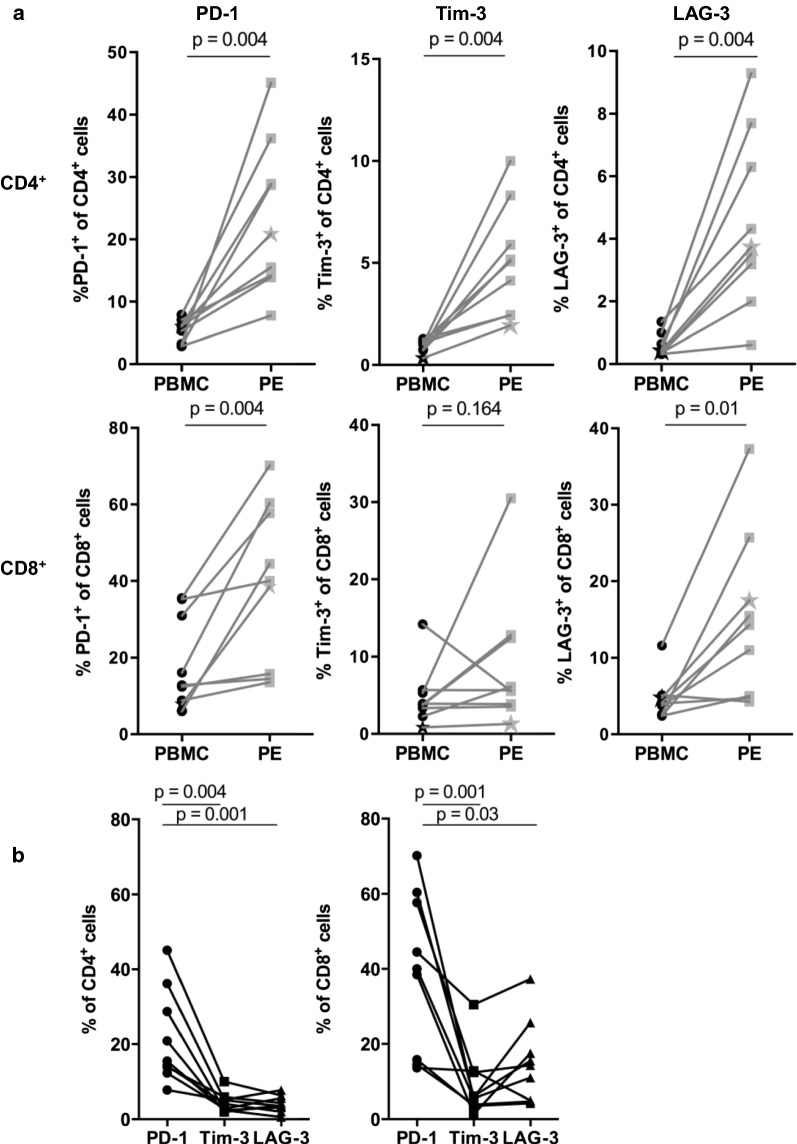
Frequency of inhibitory receptor expressing T cells in matched blood and effusion samples from malignant mesothelioma patients. **a** PD-1, Tim-3 and LAG-3 expressing CD4^+^ (top row) and CD8^+^ (bottom row) T cells plotted as a frequency of total CD3^+^CD4^+^ or CD3^+^CD8^+^ T cells respectively. Each point and connecting line represents a paired patient sample, and the star on each graph represents the single patient sample that underwent TCR sequencing. All paired samples were compared using a Wilcoxon’s test. **b** Frequency of PD-1, Tim-3 and LAG-3 expressing CD4^+^ and CD8^+^ T cells from effusion samples. Matched samples were compared with a Friedman test, corrected for multiple comparisons using Dunn’s test

T cells within the tumour environment can display a dysfunctional phenotype characterized by expression of T cell inhibitory receptors [9]. Therefore we compared the cell surface expression of PD-1, LAG-3 and Tim-3 on T cells in peripheral blood and PE. In the CD4^+^ T cell population, there was a statistically significant larger proportion of PE T cells that expressed PD-1 (p = 0.004), Tim-3 (p = 0.004) and LAG-3 (p = 0.004) compared to peripheral blood (Fig. 1a). The proportion of CD8^+^ T cells expressing PD-1 (p = 0.004) and LAG-3 (p = 0.01) was also significantly higher in the PE compared with peripheral blood (Fig. 1a). Overall, the proportion of CD4^+^ and CD8^+^ T cells expressing PD-1 in PE was five-fold higher than T cells in peripheral blood, with PD-1 being the inhibitory receptor most overexpressed in PE T cell subsets compared with Tim-3 (CD4^+^ p = 0.004, CD8^+^ p = 0.0001) and LAG-3 (CD4^+^ p = 0.002, CD8^+^ p = 0.04) (Fig. 1b).

#### Malignant effusions have diverse TCR repertoires compared to blood

We examined the T cell repertoire by sequencing the TCRβ chain in T cells from PE and matched peripheral blood. As PD-1 was the most abundantly expressed T cell inhibitory receptor in our PE samples compared with blood, T cell subsets were further sorted into CD4^+^PD-1^+^ and CD8^+^PD-1^+^ populations. We performed high throughput TCRseq in CD4^+^, CD4^+^PD-1^+^, CD8^+^ and CD8^+^PD-1^+^ T cells derived from matched PE and peripheral blood. This was performed in a single participant (patient 9 in Table 1), who had no prior treatment that might alter the TCR repertoire, and was selected on the basis of sample availability.

**Fig. 2:**
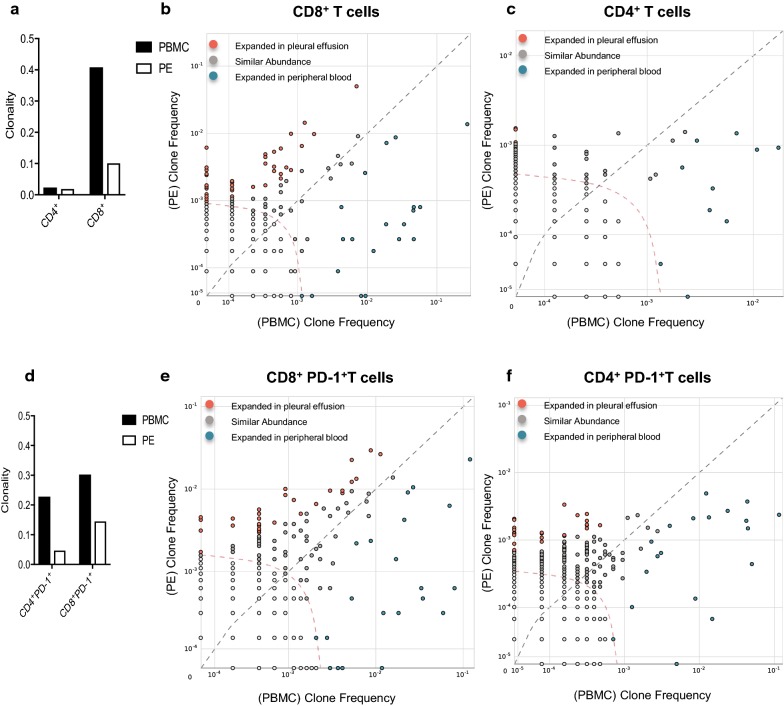
PE T cells consist of unique TCR clonotypes compared with peripheral blood. **a** Clonality score of bulk CD4^+^ and CD8^+^ populations in effusion and blood. **b** Scatter plot of the most frequent TCR clonotype abundance in PE versus PB, CD8^+^ T cells. Red circles represent 45 individual TCR clonotypes that were present at significantly higher frequencies in the effusion than in the blood. Blue circles represent twenty clonotypes at higher frequencies in the blood than PE. **c** Scatter plot of the differential abundance of TCR clonotypes in PE versus PB, CD4^+^ T cells. Ten clones present at higher frequencies in the blood, and three clones higher in PE. Dotted curve line represents threshold for statistical comparison. **d** Clonality score of PD-1^+^ T cells. **e** Scatter plot of differential abundance of TCR clonotypes in CD8^+^PD-1^+^ T cell populations. Twenty-one clones at higher frequencies in blood, thirty-one clones higher in PE. **f** Differential abundance in CD4^+^PD-1^+^ populations. Nineteen clones at higher frequencies in blood, twenty-seven clones higher in PE

CD4^+^ T cells in PE and PB demonstrated receptor diverse populations (Clonality < 0.02) (Fig. 2a). CD8^+^ T cells in blood had a higher clonality score compared to the PE, suggesting expansion of specific TCR clonotypes in the blood (Fig. 2a). A single dominant TCR clone comprised 27.6% of total CD8^+^ T cells in PB, but only 1.4% of effusion CD8^+^ T cells. Both blood and PE contained distinct CD8^+^ T cell clonotypes that differentially populated one compartment or the other (Fig. 2b). There were fewer differentially expressed CD4^+^ clonotypes (Fig. 2c).

When we examined the PD-1^+^ subsets, the clonality scores in the blood were similarly higher than the effusion (Fig. 2d). The number of differentially expressed CD8^+^PD-1^+^ clones in each compartment were similar to the CD8^+^ analysis (Fig. 2e). Interestingly, the highly dominant CD8^+^ T cell clone in this patient’s blood was not present in the CD8^+^PD-1^+^ PBMCs, suggesting this patient had an antecedent infection independent of the pleural space. Importantly, the number of differentially expressed CD4^+^ T cell clones in each compartment was higher in the CD4^+^PD-1^+^ analysis compared to the CD4^+^ analysis (27 vs. 3; Fig. 2f). There were marked clonotypic expansions in CD4^+^PD-1^+^ population that were not evident in the bulk CD4^+^ population, showing that some differences were only observed after fine analysis of clonal frequency associated with phenotype.

### Discussion

Tumour biopsies from patients with mesothelioma are difficult to obtain, can cause significant morbidity, and are usually only taken once at diagnosis unless a subsequent surgical resection is performed. Thus, serial collection of malignant PE provides a unique opportunity to analyze dynamic events adjacent to the tumour microenvironment over time. A recent study by Aaerts et al. showed that the cellular composition of malignant PE over time is dynamic and influenced by response to treatment [10]. While this study was limited to a small group of patients, ongoing longitudinal studies will determine if PE T cells behave similarly to TILs and peripheral blood lymphocytes during disease progression and immunotherapy. Our data suggest that PE T cells, like TILs, exhibit an exhausted phenotype characterized by increased expression of inhibitory markers as compared with peripheral blood T cells. In line with this, previous studies have demonstrated increased expression of PD-1 and Tim-3 on CD4^+^ and CD8^+^ T cells in PE compared with matched blood in patients with mesothelioma [11, 12] and lung cancer [13–15], suggesting that T cells in the PE more closely approximate TILs than do T cells in peripheral blood. Of note, the expression of co-inhibitory markers on TILs has been shown to correlate with response to immunotherapy [16], supporting the potential for PE T cells to provide predictive information longitudinally.

We examined the T cell repertoire from PE and blood in a single patient using TCRseq. CD4^+^ and CD8^+^ TCR repertoires were diverse in the PE, suggesting minimal T cell expansion in the PE of this patient. This contrasts oligoclonal T cell expansion observed in ascites derived from patients with ovarian cancer [17]. We observed a more restricted TCR repertoire in effusion CD4^+^PD-1^+^ T cells compared to CD4^+^ T cells, similar to previous studies studying PD-1^+^ TILs [9, 18]. In depth analysis of CD4^+^PD-1^+^ T cells also uncovered more differentially expressed TCR clones in the effusion, suggesting that focusing on PD-1^+^ T cells might yield important insight into the immunological milieu.

PD-1^+^ T cells is a population of interest to understand the relationship between T cells in the peripheral blood, PE and tumour. As we reported the findings from a single patient, it remains to be seen if TCR repertoire in the PE is equally diverse in other patients with mesothelioma, or if the repertoire changes after different therapies. The specificity of T cells in the effusion is still to be determined, as is their ability to recognize tumour antigens.

## Limitations

The study was limited by small patient numbers, especially with one patient for the TCR analysis. Tumour biopsies were unavailable for a comprehensive comparison of inhibitory receptor expression and TCR diversity between all compartments.
